# Conductive hydrogels: intelligent dressings for monitoring and healing chronic wounds

**DOI:** 10.1093/rb/rbae127

**Published:** 2024-11-01

**Authors:** Ying Fang, Yiran Han, Lu Yang, Ranjith Kumar Kankala, Shibin Wang, Aizheng Chen, Chaoping Fu

**Affiliations:** Institute of Biomaterials and Tissue Engineering & Fujian Provincial Key Laboratory of Biochemical Technology, Huaqiao University, Xiamen, Fujian 361021, P. R. China; Institute of Biomaterials and Tissue Engineering & Fujian Provincial Key Laboratory of Biochemical Technology, Huaqiao University, Xiamen, Fujian 361021, P. R. China; Institute of Biomaterials and Tissue Engineering & Fujian Provincial Key Laboratory of Biochemical Technology, Huaqiao University, Xiamen, Fujian 361021, P. R. China; Institute of Biomaterials and Tissue Engineering & Fujian Provincial Key Laboratory of Biochemical Technology, Huaqiao University, Xiamen, Fujian 361021, P. R. China; Institute of Biomaterials and Tissue Engineering & Fujian Provincial Key Laboratory of Biochemical Technology, Huaqiao University, Xiamen, Fujian 361021, P. R. China; Institute of Biomaterials and Tissue Engineering & Fujian Provincial Key Laboratory of Biochemical Technology, Huaqiao University, Xiamen, Fujian 361021, P. R. China; Institute of Biomaterials and Tissue Engineering & Fujian Provincial Key Laboratory of Biochemical Technology, Huaqiao University, Xiamen, Fujian 361021, P. R. China

**Keywords:** conductive hydrogels, intelligent dressing, chronic wound, electrical stimulation, real-time monitoring

## Abstract

Conductive hydrogels (CHs) represent a burgeoning class of intelligent wound dressings, providing innovative strategies for chronic wound repair and monitoring. Notably, CHs excel in promoting cell migration and proliferation, exhibit powerful antibacterial and anti-inflammatory properties, and enhance collagen deposition and angiogenesis. These capabilities, combined with real-time monitoring functions, play a pivotal role in accelerating collagen synthesis, angiogenesis and continuous wound surveillance. This review delves into the preparation, mechanisms and applications of CHs in wound management, highlighting their diverse and significant advantages. It emphasizes the effectiveness of CHs in treating various chronic wounds, such as diabetic ulcers, infected wounds, temperature-related injuries and athletic joint wounds. Additionally, it explores the diverse applications of multifunctional intelligent CHs in advanced wound care technologies, encompassing self-powered dressings, electrically-triggered drug delivery, comprehensive diagnostics and therapeutics and scar-free healing. Furthermore, the review highlights the challenges to their broader implementation, explores the future of intelligent wound dressings and discusses the transformative role of CHs in chronic wound management, particularly in the context of the anticipated integration of artificial intelligence (AI). Additionally, this review underscores the challenges hindering the widespread adoption of CHs, delves into the prospects of intelligent wound dressings and elucidates the transformative impact of CHs in managing chronic wounds, especially with the forthcoming integration of AI. This integration promises to facilitate predictive analytics and tailor personalized treatment plans, thereby further refining the healing process and elevating patient satisfaction. Addressing these challenges and harnessing emerging technologies, we postulate, will establish CHs as a cornerstone in revolutionizing chronic wound care, significantly improving patient outcomes.

## Graphical abstract

**Figure:**
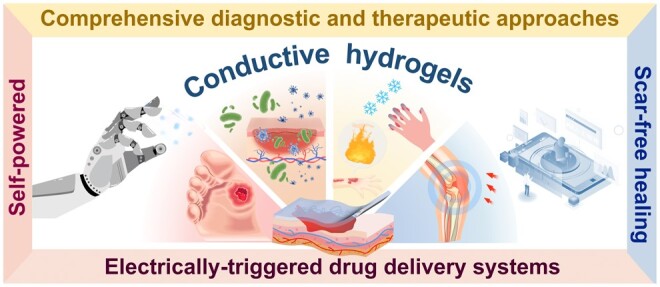

## Introduction

Trauma holds a significant position in the worldwide mortality spectrum. According to the data provided by the World Health Organization (WHO), someone dies from a car accident every 50 s, and someone dies from trauma every 2 s worldwide [1]. Hence, promoting efficient and expedited wound healing is a pivotal public health and safety concern.

Despite the skin's natural regenerative ability, inadequate wound care can impede healing. Proper wound management is crucial to prevent infection, maintain moisture, reduce pain, accelerate healing and minimize scarring [2]. Traditional hemostatic materials exhibit limitations in addressing diverse wound types and stages of healing. The Food and Drug Administration (FDA) has approved several products, but they often prove ineffective for injuries involving internal bleeding or vital tissue damage [3, 4]. Removing bandages and gauze post-hemostasis risks secondary injury, compromising the healing process. Intelligent wound dressings enabling regular monitoring of symptoms like redness, swelling, pain and exudates are invaluable for assessing healing progress and identifying infection risks [5]. Winter postulated in 1962 that a moist wound environment might facilitate rapid and improved healing [6–8]. Polymer hydrogels, with their hydrophilic porous structure, effectively absorb surplus moisture and mimic the natural extracellular matrix (ECM) structurally [9]. They provide moisture, maintain an environment conducive to cell migration, offer a cooling effect to reduce pain and are suitable for irregular and large wounds requiring frequent dressing changes without causing secondary damage [10]. Extensive research in skin cell biology, polymer scaffolds and tissue regeneration has led to commercial medical products tailored for skin tissue engineering. Recently, soft dressings leveraging hydrogels' hydrophilicity, high water content, soft-tissue-like texture, biocompatibility and flexibility have emerged for skin wound treatment [11].

The hydrogel material utilized in wound management can soak up hundreds to thousands of times its weight in water and promptly stabilize into a gel-like structure upon contact. With superior elasticity and swelling capacity, hydrogels maintain solid stability while exhibiting the fluidity essential for wound management. Due to these unique properties, hydrogels blend solid stability with liquid fluidity, leading to their extensive use in wound management. Both natural polymer hydrogels (e.g. peptides, polysaccharides, proteins, DNA, etc.) and synthetic hydrogels [e.g. polyacrylamide (PAM), polyvinyl alcohol (PVA), polymethacrylates, etc.] have better applications in biomedical fields such as tissue engineering, wound healing and drug delivery. Among them, bioengineered composites consisting of decellularized matrices with different types of biomaterials offer good prospects for tissue repair [12]. While hydrogel dressings are capable of maintaining wound moisture and promoting healing, they have limitations such as limited application scenarios, inadequate bacterial barriers and insufficient mechanical properties.

In recent years, the swift advancement of stretchable and flexible electrodes in soft electronics has facilitated using hydrogel-based systems as implantable biomedical devices, commonly referred to as bioelectronics [13–16]. Conductive hydrogels (CHs) have emerged as prevalent soft materials in biomedical engineering due to the integrated advantages of hydrogels with the physiological and electrochemical features of conductive materials. This fusion positions CHs as an excellent medium for enhancing electrically sensitive tissue regeneration [17]. Their unique 3D porous structure, hydrophilicity and tunable chemical and physical attributes qualify them as a critical substrate for cellular growth, migration and multiplication [18]. Additionally, their multifunctionality opens promising avenues for long-term clinical applications in tissue restoration. Employing CHs spreads electrical currents uniformly across wounds and mitigates the hazards linked to high-voltage electrical stimulation (ES). With reasonable design, CHs could potentially emerge as a versatile bioelectronic material meeting diverse requirements.

**Figure 1.**
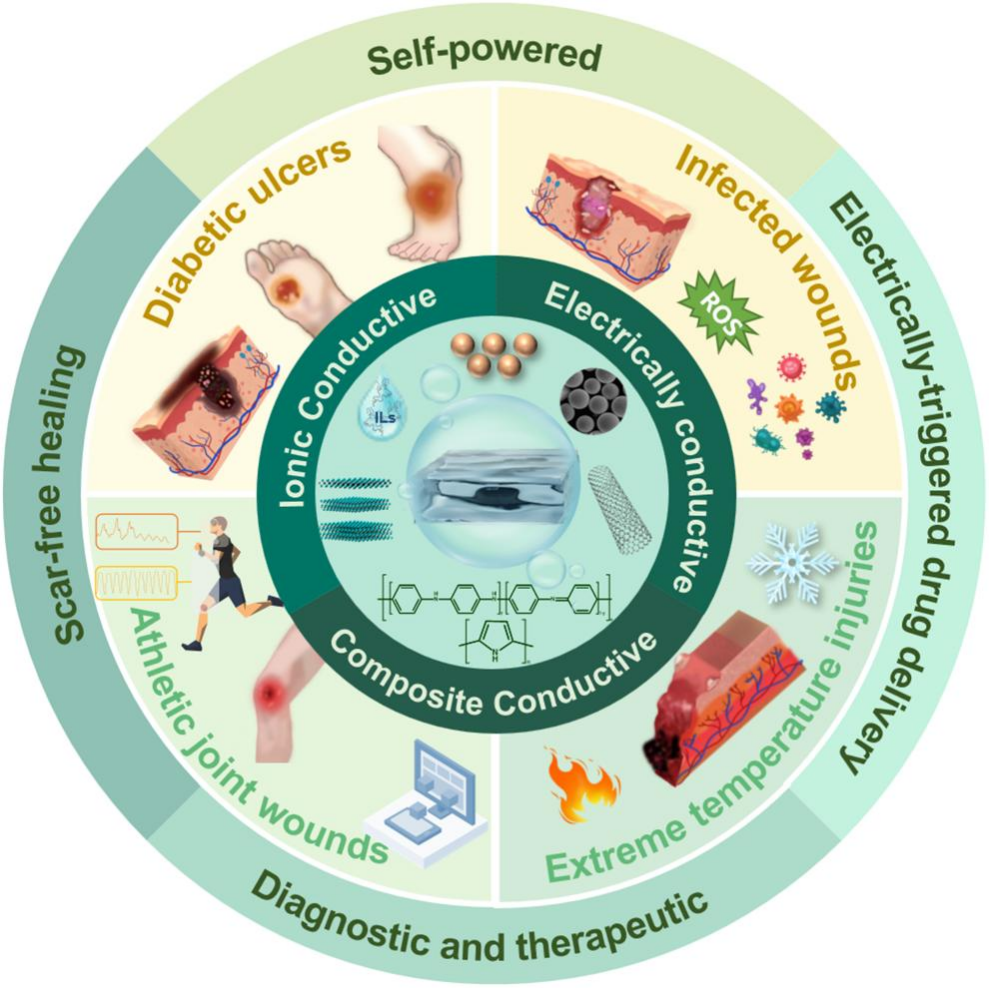
Overview of CHs types and their applications in wound healing. This figure categorizes CHs based on their composition and conductive properties and maps their specific uses across various wound types.

In this review, we examine the preparation techniques and structural attributes of CHs, focusing on their role as intelligent dressings for continuous monitoring of chronic wound healing. We summarize the preparation of CHs and their mechanism of action in wounds in terms of their antibacterial and anti-inflammatory properties, promotion of cell migration and proliferation, and acceleration of angiogenesis. We also analyse their personalized monitoring and application in managing various chronic wounds, including diabetic ulcers, infected wounds, extreme temperature injuries and athletic joint wounds (as shown in Figure 1). This article emphasizes that CHs have greater development potential and prospects in self-powered dressings, electrically-triggered drug delivery systems, comprehensive diagnostic and therapeutic approaches, scar-free healing, etc. CHs demonstrate considerable potential for advancement in the field of intelligent wound dressings. It is anticipated that these advanced materials will converge with artificial intelligence (AI), to advance personalized medical solutions, optimize healthcare outcomes and bolster human wellness.

## Preparation and structure of CHs

CHs are typically formulated by mixing different conductive materials. Standard preparation techniques involve the introduction of hydrophilic crosslinking agents into conductive polymer (CP) dispersions or the *in situ* synthesis of CPs within the hydrogel network [19]. However, integrating conductive materials into biomaterials is challenging because many CPs, carbon nanomaterials, metals and their oxides exhibit poor water solubility and a tendency to aggregate, reducing conductivity [20–22]. To overcome these limitations, sophisticated pretreatment methods are employed to ensure uniform dispersion of these materials. Surface modification techniques have effectively improved the solubility and stability of conductive nanomaterials. In addition, polymer coating techniques, aided by vigorous stirring and ultrasonic treatment, facilitate the uniform distribution of nanoconductive materials and prevent aggregation [23]. Functionalization of CPs can also be achieved by doping. It is crucial to carefully consider the stability, degradability and cytotoxicity of CPs under physiological conditions [24]. Carbon and metal nanomaterials exhibit poor biodegradability *in vivo*, which limits their applicability in certain scenarios [25]. Furthermore, the introduction of conductive materials can significantly alter the mechanical properties of the original biomaterial [26]. Therefore, a comprehensive evaluation of the balance between conductivity, biocompatibility and mechanical properties is imperative before *in vivo* application of CHs. Based on their conduction mechanisms, CHs can be broadly categorized as electronic conductors or ionic conductors, which facilitates a profound understanding of their properties and potential applications. A comparison of the performance advantages and disadvantages of CHs with different conductive compositions is shown in Table 1.

**Table 1.** Comparison of advantages and disadvantages performance of CHs with different conductive compositions

Types	Components	Conductivity^a^	Mechanical strength^a^	Biocompatibility^a^	Advantages	Disadvantages	Ref
Electronic CHs	Carbon-based CHs	Marginally higher	Depends on the morphology of the carbon material and the crosslink density of the hydrogel	Good	Antibacterial and drug-loading characteristics	Insufficient stability and tendency to aggregate	[27]
	CPs-based	Depends on the type of polymer and level of doping	Depends on the type of polymer and doping level	Poor	Electrical conductivity similar to skin	Biodegradability require enhancement	[28]
	Metal/metal oxide nanoparticle-based CHs	Marginally higher	Marginally stronger	Depends on the type of metal or metal oxide	Nanoparticle-mediated antibacterial performance Tunable performance through nanoparticle size adjustment	Metal ions may induce allergic reactions	[29]
Ionic CHs	Metal salt ionic CHs	Lower, but can be optimized by adjusting salt concentration	Weaker	Better	Self-healing ability Ion-concentration-regulated electrical conductivity	Humidity-dependent conductivity limits dry wound application	[30]
	Ionic liquid CHs	Marginally higher	Marginally stronger	Poor	Low vapor pressure and good thermal stability	Biocompatibility concerns exist	[31]
Composite CHs	Integrate ionic and electronic	Significantly higher	Highly robust	Depends on the specific materials used and their interactions	Combined electronic and ionic conduction Customizable for specific wound types	Imbalance in electron and ion concentration prone to aggregation	[32]

a The above comparisons are based on general trends and are not hard and fast rules. Actual performance may vary depending on specific material formulation, processing methods and test conditions.

### Electronic CHs

The conductivity of electronic CHs is primarily due to the directional movement of free electrons within the hydrogel system. These hydrogels are typically synthesized by incorporating electronically conductive nanoparticles (NPs), such as argentum (Ag) nanowires, graphene and carbon nanotubes (CNTs), into the hydrogel matrix. These NPs form a hybrid network within the polymer matrix, enhancing mechanical properties and translating conformational shifts in surface-bound stimulus-responsive polymers into discernible changes in properties. In addition, conducting polymers (CPs) such as polyaniline (PANI) [33], polypyrrole (PPy) [34–37] and poly(3,4-ethylenedioxythiophene) (PEDOT) [38] can serve as monomers for the construction of CHs networks. These polymers possess a π-conjugated structure with alternating single and double covalent bonds, facilitating electron conduction and solvent ion transport, ultimately resulting in efficient conduction within the hydrogel network.

#### Carbon-based CHs

**Figure 2.**
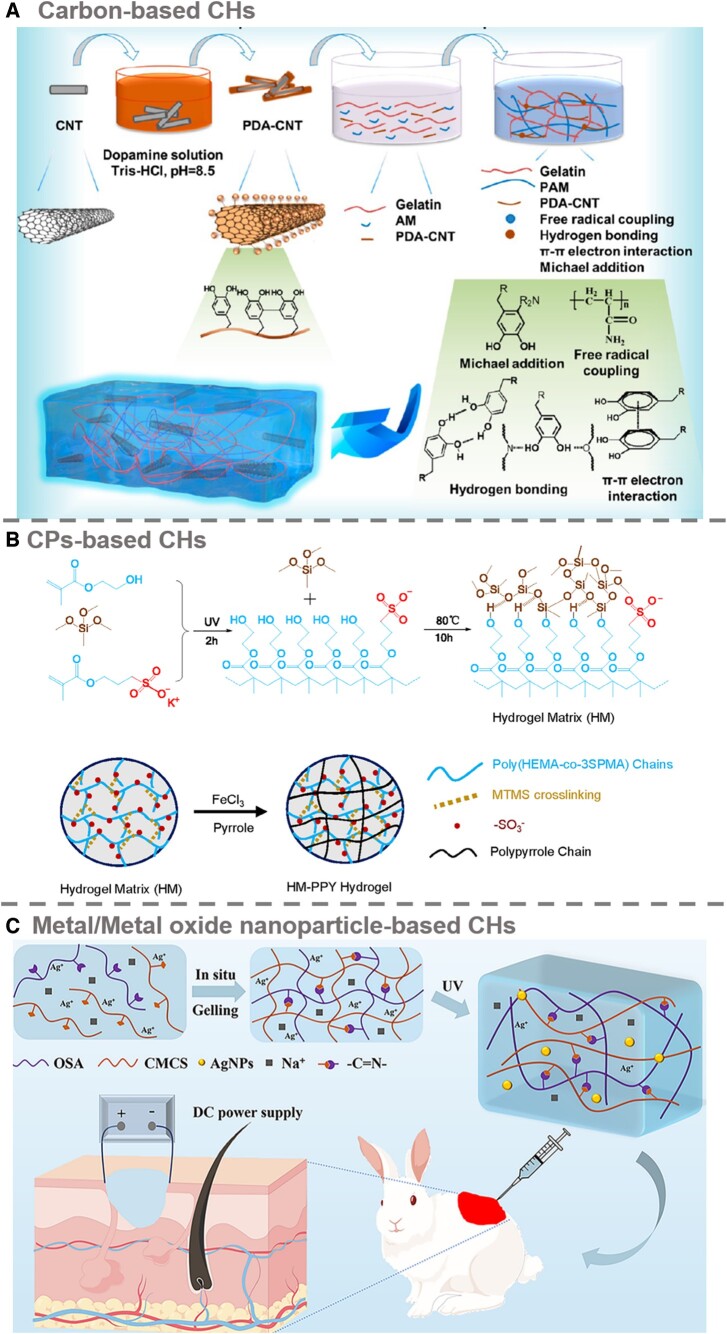
Types of electronic CHs: (**A**) Carbon-based CHs: PDA@CNTs were used as the conductive component, and flexible, biocompatible and electrically conductive all-in-one adhesive hydrogels were produced by crosslinking a mixture of gelatin and acrylamide, which are capable of both detecting human motion and accelerating wound healing through electrical stimulation generated by piezoelectricity. Reprinted from Ref. [39]. Copyright 2023, American Chemical Society; (**B**) CPs-based CHs are electrically CHs: an electrically conductive poly(2-hydroxyethyl methacrylate) (poly HEMA)/PPy hydrogel was prepared, and the electrically conductive nature of the hydrogel in a weakly alkaline physiological environment was preserved by *in situ* doping of the PPy, allowing its application in the treatment of chronic diabetic wounds with electrical stimulation. Reprinted from Ref. [35]. Copyright 2023, Elsevier; (**C**) Metal/metal oxide NPs-based CHs: argentum nanoparticles as a conductive component in a hydrogel prepared by crosslinking sodium alginate oxide and carboxymethyl chitosan and applied to full-thickness cortical defect wounds. Reprinted from Ref. [29]. Copyright 2023, Elsevier.

Carbon-based materials, including CNTs, graphene oxide (GO), activated carbon, carbon dots (CDs) and MXene, exhibit robust mechanical properties and superior conductivity, rendering them highly suitable for converting hydrogels into conductive frameworks. Notably, CNTs and GO have been extensively incorporated into CHs. CNTs, particularly in single- and multi-walled varieties, stand out as a prevalent conductive carbon material and have undergone extensive research across multiple domains (as shown in Figure 2A) [39]. In skin tissue engineering, CNTs have exhibited promising features, including antibacterial properties, efficient drug loading, enhanced angiogenesis and the ability to manipulate gene expression related to tissue regeneration [27]. A simple technique allows the use of 1D multi-walled CNTs and 2D hydroxylated hexagonal boron nitride (BN-OH) as thermally conductive additives to form 3D electrically conductive structures within a hydrogel, specifically for use in cooling therapies and burns treatment [40]. It is worth noting that CNTs are inherently hydrophobic and challenging to disperse in aqueous environments, necessitating hydrophilic modifications. One such method to reduce hydrophobicity involves using polydopamine (PDA). Li and colleagues [41] crafted a multi-purpose electronic skin, leveraging dopamine-modified CNTs and PVA. Within this material, CNTs significantly impacted the electromechanical and mechanical properties of the hydrogel, while PDA provided robust and repeatable adhesive qualities. The end product was a highly sensitive hydrogel capable of detecting subtle mechanical changes associated with human movement. Furthermore, this hydrogel could function as a conductive bridge for transmitting electrical impulses generated by a poly(vinylidene fluoride-tetrafluoroethylene) piezoelectric film, potentially aiding wound-healing acceleration. Nonetheless, given that the reported toxicity of CNTs varies based on factors like length, diameter, impurities and modifications, their cytotoxicity must be rigorously evaluated when in direct contact with human cells [42].

MXenes (Ti_3_C_2_T_*x*_), 2D materials composed of transition metal carbides and carbonates, have gained significant attention in biomedical applications alongside other 2D materials [32, 43]. These MXenes exhibit numerous compelling attributes, including hydrophilicity due to surface functional groups like hydroxyl, oxygen or fluorine, excellent electrical conductivity, mechanical flexibility and notable biocompatibility. MXene also shows superior antibacterial activity against Gram-negative *Escherichia coli* (*E.coli*) and *Staphylococcus aureus* (*S.aureus*) compared to GO. These characteristics render MXene an excellent nanofiller for the creation of CHs. Within MXene-infused hydrogels, the nanosheets form robust interactions with the hydrogel matrix via their surface functional groups, establishing a 3D conductive framework. This design gives the hydrogel superior conductivity and preserves its flexibility and tunable mechanical properties. The extensive surface area and high conductivity of MXene further enhance the sensitivity and responsiveness of the hydrogel. Due to the abundance of surface functional groups (–OH, –F and –O) on the MXene nanosheet, their incorporation strengthens the polymer crosslinking network, subsequently improving the composite hydrogel's viscosity, modulus and yield stress [32]. Despite the good electrical conductivity and mechanical properties of MXene-infused hydrogels, the dispersion of MXene nanosheets can be environmentally perturbed, leading to agglomeration and hence environmental stability [44].

#### CP-based CHs

Notwithstanding the prevalent deployment of metal NPs and carbon-based materials in tissue engineering, apprehensions about long-term cytotoxicity and stability constrain their utilization in specific contexts. Conversely, CPs demonstrate remarkable properties, with a conductivity range from 0.01 to 10 mS/cm, mirroring the conductivity of natural skin tissue [28]. These involve polymerizing CP monomers within prefabricated non-CHs nanostructures or inducing gelation by blending CPs with hydrophilic polymers/monomers *via* self-assembly and crosslinking, among others. These methods offer versatility and diversity in crafting high-performance CP hydrogels. For example, PPy, which is known for its biocompatibility, could be chosen as a conductive element, and a CHs based on poly(2-hydroxyethyl methacrylate) (poly HEMA) was designed (as shown in Figure 2B) [35]. To counteract PPy's conductivity degradation in a slightly alkaline physiological environment (pH 7.4) due to dopant loss, they innovatively copolymerized the anionic monomer 3-sulfopropyl methacrylate potassium salt (3SPMA) within the hydrogel, achieving *in situ* PPy doping. The formation of covalent bonds between 3SPMA and the hydrogel effectively prevents leakage. Furthermore, PEDOT: PSS has been employed as a conductive network in aldehyde-modified protein hydrogels [45]. Viewed through the lens of non-covalent interactions and Schiff base bonds, this system's dynamism confers distinct capabilities like self-healing, shear thinning and adhesion. These pioneering applications broaden the scope of CP hydrogels and chart new avenues for their advancement in the biomedical realm.

#### Metal/metal oxide NP-based CHs

Metal and metal oxide NPs have attracted considerable attention owing to their superior electron transfer capability, adsorption properties, optical traits and catalytic efficiency. These NPs exhibit excellent manufacturability, modifiability and biocompatibility, thus excelling in using CHs for wound management applications. Ag NPs are prominent in biomaterials due to their exceptional conductivity and antibacterial properties, endorsed by the FDA. Ag NPs can be uniformly dispersed within CHs in the presence of reducing agents (as shown in Figure 2C) [29]. Moreover, Ag and Zn dots printed on polyester or cotton fabrics function as bioelectronic wound dressings, proven to enhance human keratinocytes, activate cellular behavior and accelerate wound healing by generating continuous micro-potential and restoring physiological bioelectrical signals. The voltage varies with dot size and dot spacing [46].

### Ionic CHs

Infusing numerous free ions into the 3D polymer matrix of hydrogels concurrently imbues them with superior ionic conductivity traits. Electronic CHs often transmit intermittent electrical signals along uneven and difficult-to-distribute pathways, whereas ionic signaling forms the backbone of continuous communication in biological systems. Conversely, conductive ionic hydrogels with homogenous ion dispersal mimic intracellular ion transport, offering a persistent conductive environment for skin lesions and facilitating cell migration and proliferation. Materials capable of supplying free ions to hydrogels primarily fall into three classes: acids (HCl and H_2_SO_4_), metal salts (NaCl and LiCl) and ionic liquids (1-ethyl-3-methylimidazolium chloride). Presently, CHs wound dressings predominantly incorporate metal salts and ionic liquids (ILs).

#### Metal salt ionic CHs

**Figure 3.**
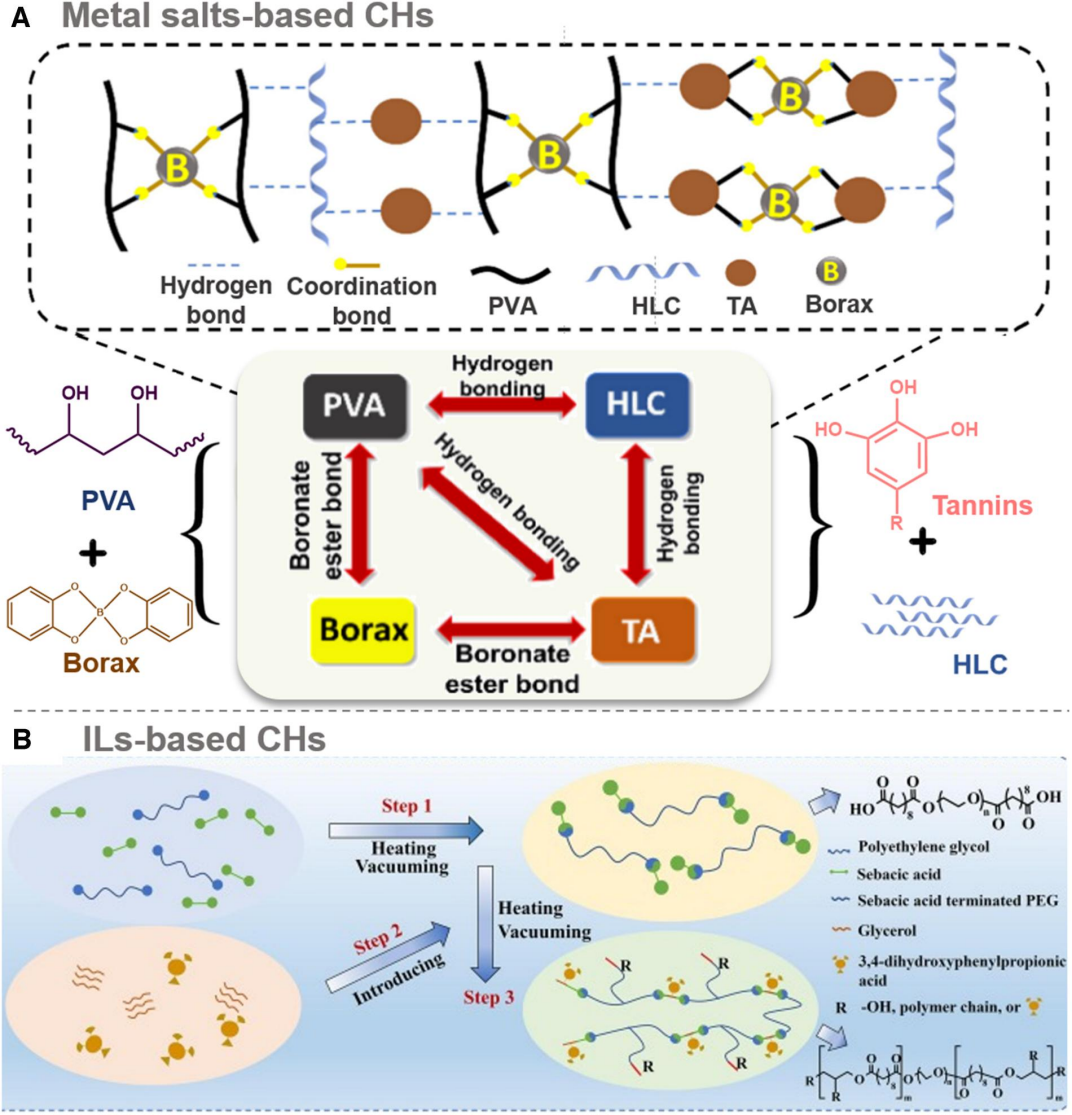
Types of ionic CHs: (**A**) Metal salts-based CHs: a conductive hydrogel for adaptive deep wounding was prepared by dynamic crosslinking of PVA and borax, doped with TA and HLC, using borate ions as the conductive component. Adapted with permission from Ref. [30]. Copyright 2021, Elsevier; and (**B**) ILs-based CHs: a highly stretchable, self-healing ILs-based CHs adhesive with antibacterial and antioxidant properties, promoting methicillin-resistant *Staphylococcus aureus*-infected motion wound healing. Reprinted from Ref. [31]. Copyright 2023, Elsevier.

Researchers have successfully formulated multiple ionically crosslinked CHs using electrostatic interaction principles. These hydrogels are created by dissolving metal salts within the hydrogel matrix, enabling metal salt ions to move through the network and confer conductivity [47]. A prime example is the hydrogel formed through ion-metal coordination between metal salt ions and carboxyl polymers. Carboxyl-containing polymers like alginate, hyaluronic acid (HA) and PVA can cooperate with metal salt ions such as sodium, potassium and magnesium. Preparing these CHs involves a straightforward process: mixing a metal salt solution with a hydrophilic polymer solution, crosslinking and gelation. These salts offer excellent solubility in water, supplying abundant ions for conductivity. For instance, crosslinking PVA and borax is a widely used method for creating ionically CHs. Based on this, Lei and colleagues [30] skillfully devised an ionically CHs named PBST by combining PVA, borax, tannic acid (TA) and human-like collagen (HLC) (as shown in Figure 3A). In this formulation, borax rapidly dissolves in water, dissociating into trigonal planar boric acid B(OH)_3_ and tetrahedral borate ions B(OH)_4_^−^. This reversible reaction allows rapid interconversion between the two forms in solution. Additionally, the decomposition of borax in water releases borax salt and sodium ions, enhancing the hydrogel's conductivity. These CHs can conform seamlessly to the contours of deep wounds, facilitating unimpeded conduction of both endogenous and exogenous currents. This attribute further promotes intercellular signaling and current transmission, positively influencing wound healing.

#### IL CHs

Despite metal salt ion CHs having a place in conductivity applications, they face limitations in conductivity, stability and biocompatibility. Conversely, IL CHs exhibit notable advantages in these critical areas. ILs, ionic compounds in a liquid state at room temperature composed of cations and anions, possess superior conductivity and thermal stability and exhibit remarkable ion mobility. Integrating ILs with hydrogels yields a novel material that outperforms metal salt ion CHs in conductivity characteristics (as shown in Figure 3B) [31]. The conductivity limitations of metal salt ions, constrained by their ion migration speed and stability, render them unsuitable for high-performance applications [48]. In contrast, ILs, characterized by their low vapor pressure and excellent thermal stability, guarantee the doped hydrogels' consistent conductivity across diverse environmental conditions. Liu and colleagues [49] adeptly blended poly(PBAimBF_4_) with oxidized hyaluronic acid (OHA) solution, successfully synthesizing a multifunctional CHs dressing utilizing polymeric ILs and konjac glucomannan (KGM) *via* Schiff base bonding between amino and aldehyde groups. This groundbreaking accomplishment further broadens the utilization of IL CHs in the biomedical domain. Nonetheless, it is crucial to acknowledge that the performance and stability of IL CHs can be affected by environmental factors, including temperature, pH and ionic strength. Hence, a comprehensive consideration of these variables is essential in practical implementations to ensure optimal performance and maintain the material's high reliability. With ongoing research and technological progress, IL CHs are poised to showcase their distinctive benefits and expansive application potential in numerous sectors.

### Composite CHs

Composite CHs integrate ionic and electronic conductivity, utilizing a polymeric matrix and conductive fillers, both ionic and electronic conductors. Their preparation often requires the simultaneous introduction of ionic and electronic conductors into the hydrogel matrix, which can be achieved by mixing, *in situ* polymerization or self-assembly techniques. Li and colleagues [32] adeptly merged an antibacterial Ag NP-coated MXene (AgNPs/MXene) nanosheet network with guar gum and phenylboronic acid-grafted sodium alginate (Alg-PBA) polymer networks, yielding a self-healing, injectable and antibacterial MXene hydrogel. Conductivity is enhanced due to the combined ionic conductivity of AgNPs and the electronic conductivity of MXene nanosheets endow the hydrogel with remarkable conductive properties. While MXene facilitates electron-based conductivity, an excessive nanosheet content can lead to aggregation, impeding sensing responsiveness by restricting sliding opportunities. Thus, regulating the ratio and concentration of electrons and ions is a crucial step in the preparation and structural design of composite CHs.

## The role of CHs in wound intelligence healing processes

**Figure 4.**
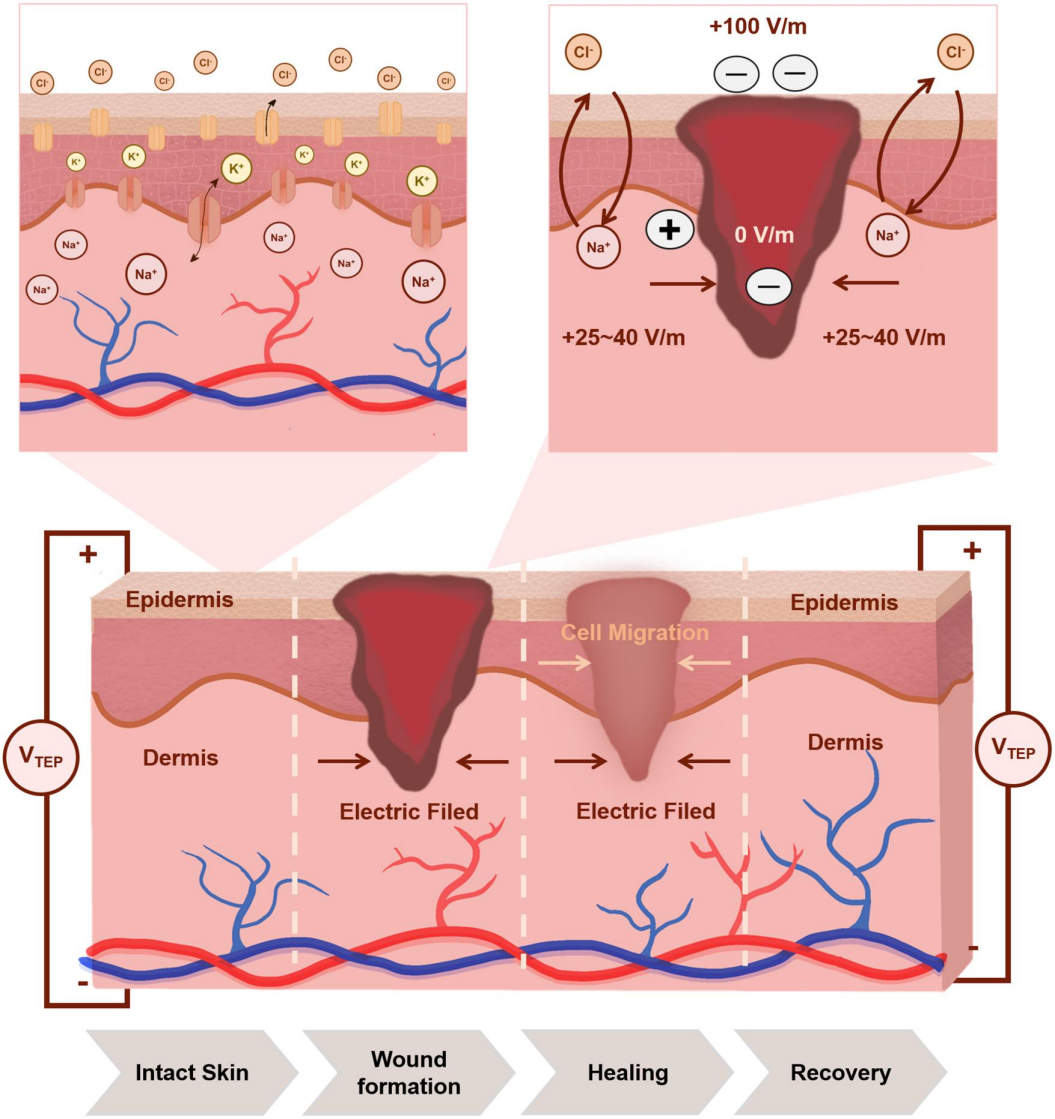
Transepithelial potentials and EF at the wound site before and after healing.

Being highly sensitive to electrical signals, skin typically exhibits conductivity ranging from 1.0 × 10^−4^ to 2.6 mS/cm [3]. Upon skin injury, an endogenous electric field (EF) emerges, distinguished by a negative polarity at the wound's center and a positive polarity at its periphery. However, specific wound conditions can impede charge transfer within this endogenous EF, thereby impeding the healing progression. Physical, ES and conductive wound dressings have been introduced to overcome this impediment, facilitating more efficient charge transfer at the wound site. Notably, Emil du Bois-Reymond first observed the presence of a direct current EF at wounds in the 19th century. Professor Min Zhao's 2006 simulation of a biological direct current EF *in vitro* revealed that various cell types, including epithelial cells and fibroblasts, migrate along the current [50]. Thakral's 2013 meta-analysis of 21 randomized clinical trials concluded that ES accelerates wound healing with minimal adverse effects [51]. The EF plays a pivotal role in directing cell migration during the healing process, highlighting the potential of electroactive scaffolds in maximizing skin tissue engineering compared to other techniques (as shown in Figure 4). Among these materials, CHs represent one type of electroactive material that can be effectively used for this purpose [52]. This discovery underscored the pivotal role of electrical signals in wound healing and laid the groundwork for novel wound treatment materials, such as CHs.

**Figure 5.**
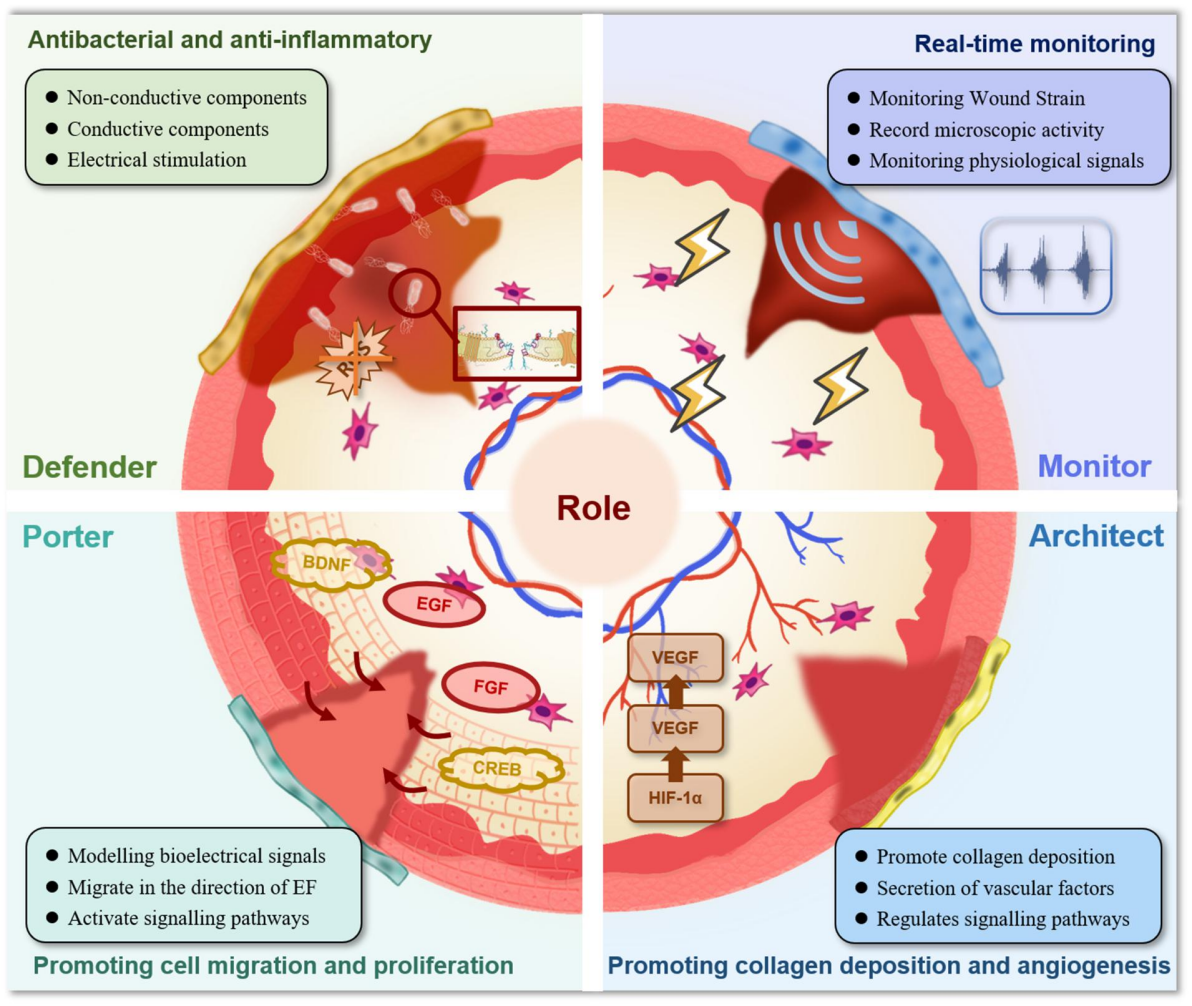
The diverse roles played by CHs in the wound-healing process, encompass their functions as defenders against bacterial invasion and facilitators of anti-inflammatory inhibition, porters enabling cell migration and proliferation, architects orchestrating collagen deposition and angiogenesis, and monitors providing real-time monitoring of the healing progress.

Owing to their electrotaxis properties, CHs dressings have received considerable attention in wound-management research. These dressings exhibit sensitivity toward detecting faint electrical currents at the wound site, actively engaging in wound healing and tissue regeneration based on these currents [53]. Functioning similarly to ‘electronic skin’, these dressings establish an optimal healing environment for wounds and notably enhance cell migration and proliferation through ES [54]. Additionally, they regulate growth factors (GFs) at the wound site, demonstrating antibacterial and anti-inflammatory properties while vigorously stimulating angiogenesis [55]. Beyond their role in facilitating uniform ES, CHs dressings offer a remarkable feature: converting wound site physiological data into interpretable electrical signals. This capability provides a valuable real-time human health monitoring tool, marking a significant advancement. Owing to their exceptional wound-healing abilities and potential for expanded functionalities, CHs dressings are increasingly becoming a focal point in wound management research. CHs are multifaceted in the context of wound care, performing a range of beneficial functions. These include enhancing cell migration and proliferation, guarding against infections, reducing inflammation, stimulating collagen and blood vessel growth, and monitoring wound status for precise healing, which in turn facilitates efficient tissue regeneration (Figure 5).

### Porter: promoting cell migration and proliferation

**Figure 6.**
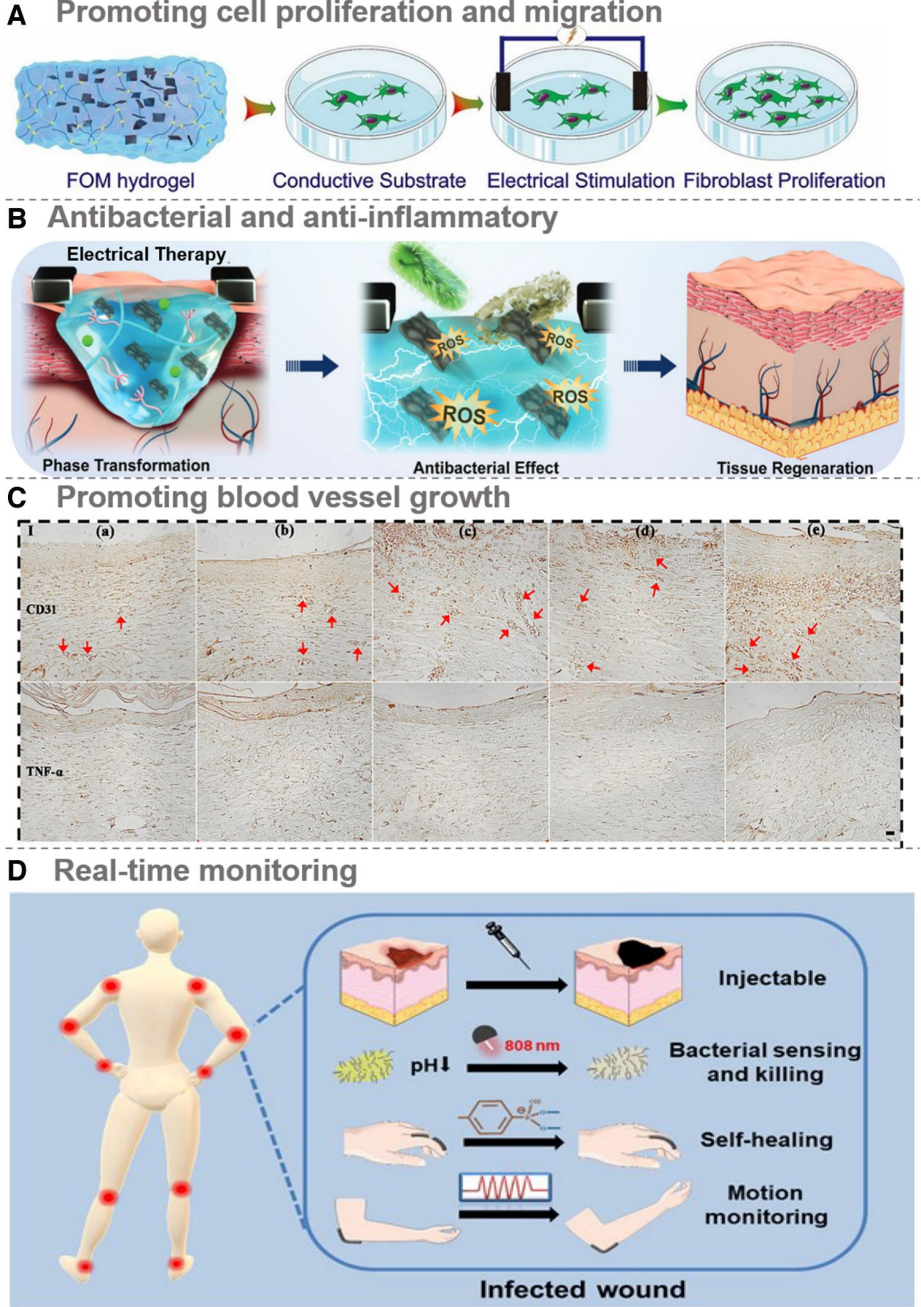
Wound repair by CHs: (**A**) Promoting cell proliferation and migration: the MXene@CeO_2_ nanocomposite multifunctional hydrogel FOM scaffolds. ES has been observed to enhance fibroblast cell mobilization and proliferation within these scaffolds. Reproduced with permission from Ref. [56]. Copyright 2021 © Elsevier; (**B**) Antibacterial and anti-inflammatory: black phosphorus-based CHs (HA-DA@BP) capable of releasing black phosphorus under mildly acidic conditions were prepared by amidation reaction and Fe^3+^ catechol coordination for synergistic electro-antimicrobial effects and wound healing promotion. Reproduced with permission from Ref. [57]. Copyright 2021 © John Wiley and Sons; (**C**) Promoting blood vessel growth: xanthan gum and silk fibroin-based burn dressings, demonstrated notable efficacy in stimulating dermal collagen matrix formation and promoting blood vessel growth in a rat model. Reproduced with permission from Ref. [58]. Copyright 2024 © Elsevier and (**D**) Real-time monitoring: the hydrogel matrix, crafted from a combination of aminobenzene boronic acid-grafted sodium alginate, PVA and hydroxylated graphene *via* dynamic and supramolecular interactions, offers exceptional properties such as bacterial infection detection through electrical signal variations, making it an ideal dressing for bacterial diagnosis, treatment and wound health management. Reproduced with permission from Ref. [59]. Copyright 2024 © John Wiley and Sons.

As previously noted, skin injuries trigger the formation of an endogenous EF oriented from the wound perimeter toward its center. This promotes the proliferation and migration of host cells, thereby fostering tissue expansion and epithelial restoration. CHs can mimic bioelectric signals by utilizing their conductivity to shape and modulate cellular dynamics. They enhance neuronal differentiation, cell growth, migration and physiological processes while bolstering intercellular communication and expediting nerve repair and regeneration. Zheng and colleagues [56] explored the impact of ES on fibroblast proliferation (Figure 6A). Their results demonstrated a notable increase in the cell count within the CHs coupled with ES (set at 600 mV) compared to the control groups, indicating that this combination stimulated cell growth. These findings using CHs as a medium implied that ES could direct cells to migrate toward damaged regions along the EF gradient, driving cell proliferation and differentiation, as well as ultimately accelerating the healing process. Recent experimental results have indicated a potentially more significant role for bioelectric signaling than previously envisioned. Illustratively, Zhao and colleagues [60] discovered that endogenous EF could modulate cell migration during tissue repair by influencing pivotal signaling cascades like phosphatidylinositol 3-kinase/protein kinase B (PI3K/Ptcn), membrane GF receptors and integrins. ES could amplify cell growth and expedite wound healing by activating the TGFb1-ERK-NF-κB (transforming growth factor-β1-extracellular regulated protein kinases-nuclear factor-κb) pathway. Moreover, CHs can emulate ECM owing to their distinct physical and chemical traits, such as pliancy, viscoelasticity and surface wettability. These hydrogels, as scaffolds for cellular expansion, provide anchoring points that support cell growth and migration. Cell migration plays the role of a porter in intelligent wound management. It is responsible for delivering newborn cells to the wound to replace the missing tissue and lay a solid foundation for wound repair and regeneration.

### Defender: antibacterial and anti-inflammatory

Bacterial infection and inflammation significantly affect wound healing in the early stages. Effective wound healing depends on controlling bacterial growth and mitigating inflammation by eliminating reactive oxygen species (ROS) [61]. An optimal wound dressing must exhibit antibacterial properties, shielding the wound from external contaminants and eradicating bacteria at the wound site [53]. Based on their antibacterial mechanisms, they can be broadly categorized into three types: Firstly, some CHs derive their antibacterial activity from non-conductive components. TA naturally found in grapes, tea and oak trees is a prime example [62]. It acts as a defensive agent in plants, resisting fungi, insects and other organisms, and contributes to antioxidant and antibacterial functions. Studies have been conducted to enhance the antimicrobial properties of PAM/agarose hydrogels by incorporating TA-borax complexes [63]. TA inhibits biofilm formation and thus bacterial growth by interfering with interbacterial communication and manipulator-related gene expression, such as *S.aureus* and *E.coli*, thereby suppressing their growth. Second, CHs have an antibacterial effect due to the conductive components or ES. Carbon-based materials, which are frequently used as conductive elements, also exhibit antibacterial properties. For example, CNTs and rGO pierce bacterial membranes due to their nanoscale sharp edges, causing content leakage and cell death. They also produce ROS, inducing oxidative stress that disrupts membrane integrity and increases permeability [59]. Under certain conditions, these materials can generate ROS, which oxidize and kill bacteria. Li and colleagues [64] developed a multifunctional hydrogel based on quaternized chitosan (CS), PDA-coated rGO and poly(N-isopropyl acrylamide). The hydrogel's components exhibited excellent photothermal properties, enhancing its antibacterial capabilities and achieving a 100% kill rate against *E.coli* and *S.aureus*. Bacteria typically utilize flagella for movement, which rely on ion channels and pumps within the cell membrane. ES has the potential to disrupt the functionality of these structures, thereby reducing bacterial motility and, consequently, their capacity to alter their position within the biofilm and proliferate. This, in turn, can mitigate bacterial-induced inflammation. In addition, ES has been shown to produce Ag^+^ ions and ROS intermediates that can damage bacterial biomacromolecules (including lipids, proteins and nucleic acids), reduce dehydrogenase activity and induce bacterial oxidative stress [65]. This prevented biofilm formation and led to bacterial death. The antibacterial rate was shown to increase from 20% to almost 50% as the duration of ES increased (Figure 6B) [57]. The bacterial death resulting from the aforementioned process can activate the host's immune response. The damage and death of bacterial cells result in the release of cellular contents that can be recognized as pathogen-associated molecular patterns by host pattern recognition receptors, thereby activating immune cells such as macrophages and dendritic cells.

Although CHs have great potential to enhance tissue repair, their *in vivo* application may lead to a foreign body inflammatory response [66]. Persistent inflammation can hinder wound healing, whereas recurrent infections can trigger repeated cycles of inflammation. Furthermore, generating copious amounts of ROS at the wound site significantly contributes to systemic inflammation and organ dysfunction. Excessive free radicals at the wound locale can obstruct healing by inactivating enzymes, peroxidizing lipids and fragmenting DNA due to oxidative stress. So, mitigating oxidative stress through ROS scavenging offers a viable wound regeneration and healing approach. CPs, leveraging their conjugated backbone, can supply electrons for ROS scavenging, demonstrating antioxidant properties that bolster wound healing amidst inflammation [38]. In addition to CPs, carbon-based materials, and other doped elements can contribute to ROS scavenging. The standalone use of MXene was insufficient for the complete elimination of excess ROS within and around the wound, resulting in the induction of detrimental oxidative and severe inflammatory reactions [56]. To surmount this limitation, they amalgamated MXene with antibacterial NPs, CeO_2_, formulating CHs endowed with tissue adhesion, anti-inflammatory, rapid hemostasis, antioxidant and antibacterial attributes. The designed hydrogel safeguarded cells from the harmful wound microenvironment by alleviating oxidative stress and concurrently supplying oxygen. Since the inflammatory phase of wound healing transpired after the hemolytic phase, and ROS were typically expressed during the early stages, macrophages could succumb to damage from excessive ROS. Thus, a wound dressing consisting of doxycycline hydrochloride (DOXH)-loaded and ROS-degradable polyurethane (PFKU) nanofibrous membrane and 3D-printed CHs strips can be prepared; when DOXH in the hydrogel reduces ROS levels, it favors macrophage polarization toward the M2 phenotype and down-regulates the expression of inflammatory factors; and faster-releasing DOXH can modulate the inflammatory phase before ROS and inflammatory factor levels increase and impair new tissue proliferation [48]. Antimicrobial and anti-inflammatory agents play a role in intelligent wound management, acting as guardians to keep wounds clean and healthy.

### Architect: promoting collagen deposition and angiogenesis

Angiogenesis plays a pivotal role in wound healing by ensuring adequate oxygen and nutrient supply via blood flow, enhancing cell proliferation, migration and metabolic activities [67]. Collagen, enriched with arginine-glycine-aspartic acid (RGD) motifs, fosters vascular niches and stimulates angiogenesis [68].

Among the various ES, pulsed current (PC) stands out from direct current (DC) and alternating current (AC) because of its dynamic nature, adjusting frequency, pulse amplitude and pulse width during stimulation. This current effectively promotes the production of vascular endothelial growth factor (VEGF) and nitric oxide, thereby facilitating angiogenesis. According to Lin's findings [37], the application of CHs led to the enrichment of fibroblasts, myofibroblasts and neovascularization in regenerated skin tissue. The significantly smaller size of granulation tissue in the regenerated area compared to other groups suggested that the tissue induced by the CHs was highly mature, indicating a potential progression to the remodeling phase of healing. Immunohistochemical analysis highlighted the presence of large-diameter neovascularization, densely distributed mature blood vessels and a notable increase in α-smooth muscle actin and platelet endothelial cell adhesion molecule-1 (PECAM-1/CD31) expression, underscoring the efficacy of the hydrogel in promoting angiogenesis. Standard angiogenesis enhancement methods rely on delivery systems that involve cells, proteins and GFs. CHs dressings resulted in significantly higher expression of angiogenic factors at the wound site (Figure 6C) [58]. This elevation could be attributed to electronic signals transmitted by the hydrogel to the wound surface, activating cellular processes. The dressings' electroactive components may influence cellular functions *via* the calcium/calmodulin signaling pathway, activating PI3K/AKT and mitogen-activated protein kinase, kinase/extracellular regulated protein kinases (MEK/ERK) pathways. This sequence increased calcium levels, activating calmodulin in the cytoskeleton, stimulating cell proliferation and boosting VEGF and TGF-β expression. These combined effects could profoundly impact endothelial cell migration and proliferation, driving angiogenesis. Consequently, CHs, as wound dressings, exhibited multifunctional synergies, orderly collagen deposition, angiogenesis and inflammatory reaction relief, effectively expediting wound healing and enhancing healing quality. Angiogenesis and collagen deposition act as deft architects in intelligent wound management to create new lifelines for wound healing.

### Monitor: real-time monitoring

Traditional wound dressings fail to promote cellular activities crucial for wound healing and cannot monitor healing progress. However, a real-time understanding of the wound's healing status is essential for doctors and patients to assess the pathological condition precisely, enabling tailored and accurate treatments. Recently, several CHs dressings have been reported, that facilitate *in vivo* wound healing and possess strain-sensing capabilities. These dressings find application in sports-related wounds, given that body parts like the soles of feet, wrists and joints are often subjected to various pressures, stretches and twists. Any abnormalities during the healing process can hinder progress or lead to chronic wounds. Timely reporting of such abnormalities is thus crucial for effective wound management (Figure 6D) [39, 59]. For patients with severe trauma, it becomes imperative for wound dressings to have sensing capabilities to detect bodily signals promptly. When movement compresses the injured area, the hydrogel dressing must monitor the wound status in real-time to ensure optimal treatment timing, thereby reducing patient suffering and treatment duration. The CaSA-GAD hydrogel adhered to wounds while functioning as a skin sensor to detect wound strain [69]. Its high strain sensitivity was evident from comparing real-time relative current changes under different strain conditions. This hydrogel's transparency, strong adhesiveness and stable mechanical properties made it suitable for wearable electronic devices detecting human motion signals. In addition, functional epidermal strain sensors can also record various micro-human activities with rapid response times and good stability [70]. A multifunctional, stretchable hydrogel suitable for joint wound dressings has recently been reported to have self-healing, injectable, motion monitoring, *in situ* bacterial sensing and non-antibiotic killing properties [59]. This hydrogel exhibited excellent motion monitoring capabilities, maintaining a relative resistance change of approximately 20% after 600 s of 50% strain. This design prevented secondary wound disintegration in stretchable body areas. Lastly, a PVA/PAM-C1 hydrogel was prepared by combining it with a multifunctional wearable epidermal sensor to monitor physical activity. This hydrogel can sensitively detect both large body movements and subtle electrophysiological signals, providing valuable clinical insights into rehabilitation training and the management of muscle-related disorders [71]. Real-time monitoring plays the role of an information-centric monitor in intelligent wound management, making the healing process more transparent and controllable, and providing clinicians with accurate feedback to adjust the treatment plan according to the actual condition of the wound.

## Personalized management of chronic wounds with CHs

To promote the healing of wounds in a targeted manner, a comprehensive understanding of wound physiology and pathology is crucial. Wounds can be categorized into different types: acute incisional and excisional wounds heal quickly under simple conditions, while chronic wounds require specialized treatment [3]. Chronic wounds, often referred to as refractory wounds, are caused by various factors, including diabetes, prolonged bed rest, venous dysfunction, aging, surgical procedures and others. Mismanagement of these wounds can result in serious complications such as infection, amputation, disability or even death [72]. The dynamics of chronic wounds are influenced by multiple local and systemic factors, exhibiting complex individual characteristics and requiring specialized healing approaches. However, most commercially available wound dressings lack targeted therapeutic action. Hence, there is a pressing need for more precise and personalized CHs as wound dressings to address this issue (as delineated in Table 2).

**Table 2.** CHs in various scenarios and related parameters

CHs	Hydrogel substrate	Conductive component	Type of wound	Type of power supply	Conductivity (mS/cm)	Wound management mechanism	Ref
DMP/IL/PVA hydrogel	PVA	IL	Diabetic chronic wounds	DC	9.6	Promoting collagen deposition and angiogenesis Anti-bacterial and anti-inflammatory	[73]
QP-P-D hydrogel	QCS Polyethylene glycol (PEG)	PANI	Diabetic infected wounds	DC	∼0.2	Anti-bacterial and anti-inflammatory Promoting collagen deposition and angiogenesis Promoting cell migration and proliferation	[74]
PBVP hydrogel	PVA	ILs	Diabetic chronic wounds	DC	0.95 ± 0.13	Anti-bacterial and anti-inflammatory Promoting collagen deposition and angiogenesis	[75]
Hep-PDA-rGO-PAM hydrogel	PAM	Hep-PDA-rGO NPs	Diabetic chronic wounds	DC	36.3	Anti-bacterial and anti-inflammatory Promoting collagen deposition and angiogenesis Real-time monitoring	[76]
AZP-Tb hydrogel	Agarose CS	PPy	Diabetic ulcer	DC	2.90 ± 0.42	Anti-bacterial and anti-inflammatory	[77]
PIL-OHA hydrogel	OHA	ILs	Diabetic chronic wounds	AC	0.76	Anti-bacterial and anti-inflammatory Promoting cell migration and proliferation	[54]
PGCNSH hydrogel	Microcrystalline cellulose	Poly(dopamine)-reduced graphene oxide	Diabetic chronic wounds	AC	50–60	Promoting collagen deposition and angiogenesis	[78]
DCPM hydrogel	Carboxymethyl chitosan (CMCS)	M NPs CP	Diabetic infected wounds	AC	1.7	Anti-bacterial and anti-inflammatory Real-time monitoring Promoting collagen deposition and angiogenesis	[79]
HM-PPY hydrogel	Poly(2-hydroxyethyl methacrylate)	PPy	Diabetic chronic wounds	AC	∼0.8	Promoting cell migration and proliferation Anti-bacterial and anti-inflammatory	[35]
PIL-KGM hydrogel	PAM	ILs	Diabetic chronic wounds	AC	0.21–0.75	Promoting cell migration and proliferation Promoting collagen deposition and angiogenesis Anti-bacterial and anti-inflammatory	[49]
PCPZ hydrogel	PVA/CS	PPy	Infected chronic wound	DC	1.16 × 10^3^	Anti-bacterial and anti-inflammatory Promoting collagen deposition and angiogenesis	[34]
PSP hydrogel	PAM Sulfonated hyaluronic acid	PANI	Infected chronic wound	DC	1.05–1.20	Anti-bacterial and anti-inflammatory	[33]
HA-DA@BP hydrogel	HA	Black phosphorus	Bacterial infection wounds	DC	2.6 ± 0.4	Anti-bacterial and anti-inflammatory	[57]
MXene@CeO_2_ FOM	Oxidized sodium alginate (OSA)	MXene	Methicillin-resistant *Staphylococcus aureus* infected wound	AC	0.4–1.2	Anti-bacterial and anti-inflammatory Promoting cell migration and proliferation	[56]
PVA/PAA-B hydrogel	PVA Polyacrylic acid	Na^+^/(B_4_O_7_)^2-^	Full-thickness skin wound	PC	30–90	Anti-bacterial and anti-inflammatory Real-time monitoring	[80]
DC-Gel-PAM hydrogel	Gelatin PAM	PDA-CNT	Full-thickness skin wound	Piezo-electric film	1–4.95	Promoting cell migration and proliferation Real-time monitoring	[39]
OSA/CMCS/AgNPs hydrogel	OSA CMCS	AgNPs	Irregular wounds	DC	12.7	Anti-bacterial and anti-inflammatory Promoting cell migration and proliferation	[29]
PHTB hydrogel	PVA HLC	Na^+^/(B_4_O_7_)^2−^	Deep wound	DC	0.573–0.824	Anti-bacterial and anti-inflammatory Promoting collagen deposition and angiogenesis Promoting cell migration and proliferation	[30]

### Intelligent CHs for diabetic ulcers healing and blood glucose monitoring

**Figure 7.**
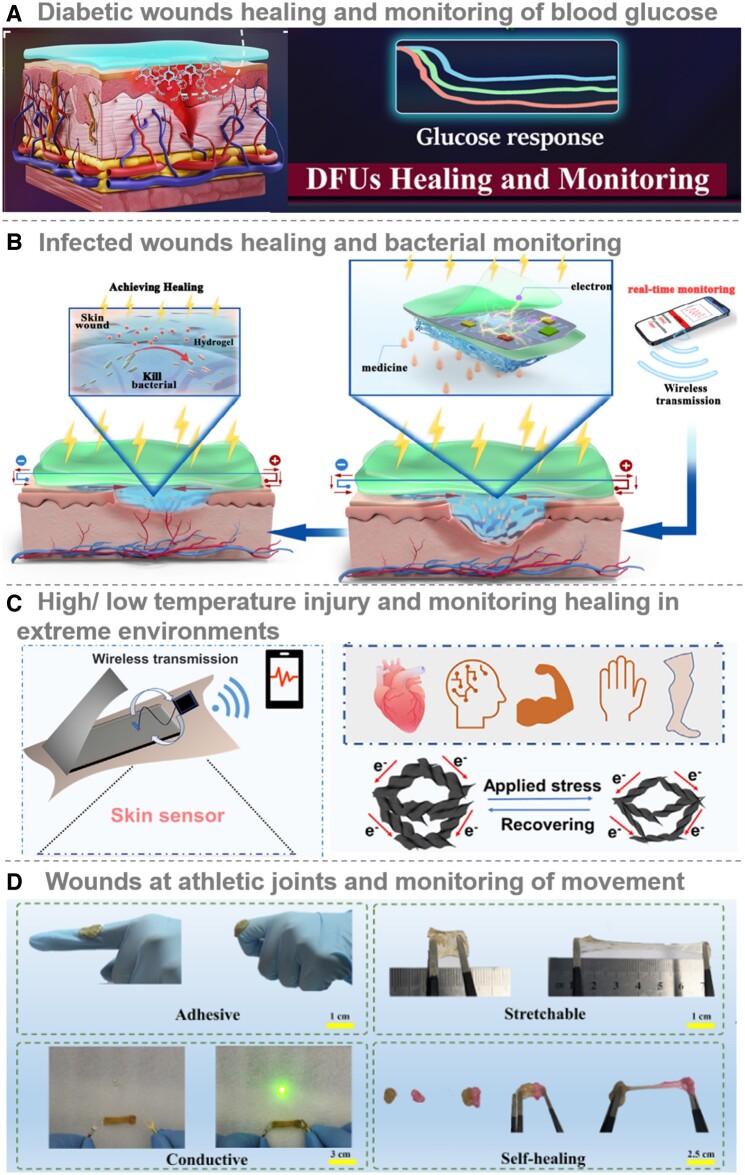
Personalized management of chronic wounds with CHs: (**A**) diabetic wound healing and monitoring of blood glucose: a highly transparent, adhesive and hemostatic CHs patch was fabricated through the *in situ* assembly of a poly(acrylamide-acrylate adenine) (P(AM-Aa)) polymer network doped with poly(tannic acid) (PTA)-infused PPy nanofibers, enabling indirect detection of glucose levels on the wound surface. Adapted with permission from Ref. [88]. Copyright 2024 © Royal Society of Chemistry. (**B**) Infected wound healing and bacterial monitoring: a self-healing QCS/OD hydrogel composed of QCS and OD that is further integrated with PVDF to form enhanced heterogeneous interfacial sensors capable of detecting infected wound gases. Reproduced with permission from Ref. [89]. Copyright 2024 © Elsevier. (**C**) High/low-temperature injury and healing monitoring in extreme environments: a multifunctional CHs composed of PAAm/PEG/hydrolyzed keratin (HK)/MXene was developed as a high-performance, integrated therapeutic epidermal sensor and its dual functionality was exemplified in a rat model of frostbite. Adapted with permission from Ref. [90]. Copyright 2023 © Elsevier; and (**D**) Wounds at athletic joints and monitoring of movement: an antimicrobial, conductive and antioxidant hydrogel adhesive with high extensibility and rapid self-healing capabilities, making it suitable for the treatment of infected sports wounds and facilitating real-time wound monitoring through its strain-sensing properties. Reproduced with permission from Ref. [31]. Copyright 2023 © Elsevier.

Diabetes is a condition characterized by either inadequate insulin production or insulin resistance, resulting in the accumulation of glucose in the bloodstream, potentially causing significant harm to essential organs [81]. As of today, there are approximately 500 million individuals worldwide diagnosed with diabetes. Notably, between 19% and 34% of these diabetic patients are prone to developing chronic diabetic wounds [82]. Diabetic wounds, primarily chronic diabetic ulcers and diabetic foot ulcers (DFUs) are severe complications of diabetes [83]. A hallmark of these wounds is the elevated levels of ROS caused by prolonged hyperglycemia. This increase in ROS leads to excessive oxidative stress, impaired antioxidant defenses and microvascular damage. In diabetic wounds, impaired angiogenesis and reduced angiogenic factors such as VEGF and platelet-derived growth factor (PDGF) prevent efficient delivery of oxygen [36], nutrients and GFs to the wound, leading to glucose accumulation and microenvironmental deterioration [84]. Diabetic wounds often stall in the inflammatory phase, with immune dysregulation and hyperglycemia increasing susceptibility to infection [72]. Prolonged high glucose levels accumulate advanced glycation end products (AGEs), disrupting the oxidative/reductive balance of the wound bed. Excessive ROS leads to lipid peroxidation, inactivation of enzymes, fragmentation of DNA and collateral damage to adjacent skin, thereby impeding wound healing [85]. Self-repairing CHs were studied by cross-linking four-armed SH-PEG with Ag^+^ and coordinating Ag-S bonds, and using ES, this hydrogel releases metformin, a drug used in the treatment of diabetes, which attenuates hyperglycemia-induced endothelial cell damage through mitochondrial autophagy [83]. Alternatively, Guan and colleagues [36] demonstrated immune modulation and nerve regeneration, integrating hydrogels with ES to harness the bioactivities of immune modulation, angiogenesis and nerve regeneration for enhanced healing of chronic diabetic wounds. Diabetic patients often suffer from peripheral vascular disease, which affects the blood supply to foot wounds. The weight-bearing function of the foot complicates wound healing. DFUs are characterized by large size, irregular shape and significant wound exudate [86]. Zhao and colleagues [87] developed a PVA-CEC-AGA/Ag hydrogel that could be readily removed because of its on-demand solubility. It provided a novel, noninvasive treatment for diabetic foot wounds while controlling glycemia and improving healing. However, current dressings limit visualization monitoring of the healing process. The development of a highly transparent CHs patch P(Py-TA)/CHA, which allows direct monitoring of the wound, can address this shortcoming (Figure 7A) [88]. This hydrogel accelerated hemostasis, improved intercellular communication, prevented infection, enhanced collagen deposition and stimulated angiogenesis. In addition, its resistance changed with glucose concentrations, allowing real-time glucose monitoring that correlated well with commercial glucometers. This integrated approach provided a reliable reference for diabetic foot management in clinical practice.

### Intelligent CHs for infected wounds healing and bacterial monitoring

Bacterial infection significantly impacts wound healing, often leading to the formation of chronic wounds. When bacteria invade a wound, the immune system initiates defense mechanisms, such as inflammatory cell infiltration, blood vessel dilation and exudate increase, to eliminate pathogens and promote healing [75, 91]. However, excessive bacterial load or highly virulent bacteria can overwhelm these defenses, resulting in wound infection. Drug-resistant bacteria, especially in multidrug-resistant infections, pose a significant threat to patient health and quality of life, reducing the effectiveness of commonly used antibiotics [56]. However, microbial infections are closely linked to changes in physiological signals within the wound microenvironment, such as blood glucose, temperature, pH, enzymes, pressure and uric acid (Figure 7B). These changes often precede clinically significant infection symptoms, making *in situ* monitoring of physiological signals a promising strategy for early infection detection, guiding dynamic therapies and effective wound management. As an intuitive physiological signal, temperature indicates wound inflammation and healing progress. The implementation of temperature monitoring facilitates the acquisition of real-time data on the extent of wound inflammation, thereby enhancing the precision and personalization of wound diagnosis. This, in turn, minimizes the risk of misdiagnosis, treatment delays and the deterioration of wounds due to untimely intervention. Jiang and colleagues [92] developed a CHs dressing capable of real-time monitoring of wound temperature changes to assess healing and infection status. This dressing, equipped with wireless transmission capabilities, converted temperature changes into data *via* a resistance-temperature converter and transmitted them to an intelligent phone *via* Bluetooth. As healing progressed, the overall wound temperature tended to decrease. pH is another vital indicator of wound damage, infection status and physiological changes during healing. Understanding wound pH aids in assessing healing and infection risks and selecting appropriate dressings and treatments. A conductive bilayer wound dressing has been developed to promote healing and enable real-time monitoring of surface temperature and pH [93]. Healthy skin maintains an acidic pH, but when injured, the pH increases due to microvascular leakage, favoring bacterial infection. Restoring an acidic environment could reduce the microbial load and susceptibility to bacterial colonization, aiding in metabolic recovery. However, keratinocyte and fibroblast proliferation favored an alkaline environment. This dressing provided valuable insights into dynamic wound environment changes, enabling early complication detection and timely interventions for optimal healing.

Besides indirect monitoring of physiological signals, Nhien and colleagues [94] innovatively merged wound healing metrics with infection status determination by employing CHs formulated from PDA-crosslinked CMCS and an interlaced array electrode. This hydrogel dressing could sense the resistance or output current of the wound tissue, facilitating noninvasive, continuous tracking of the entire healing process. The wound recovery index (RI) allowed for accurate and noninvasive assessment of healing progression. Upon infection with *E.coli*, the RI experienced a notable decline. Consequently, a persistently low RI prompted a real-time warning signal on the intelligent phone *via* the WIFI system, immediately notifying patients and medical staff of potential wound infection and enabling swift intervention.

### Intelligent CHs for extreme temperature injuries and monitoring healing

Extreme temperatures can significantly impact human skin, often resulting in wound damage. Specifically, exposure to very low temperatures may cause frostbite, leading to wound formation, whereas excessively high temperatures can induce burns. Burns, classified as a severe thermal injury, carry substantial risks, including wound infection, sepsis and potential multi-organ system complications [95]. Such injuries can extensively damage potassium transporters and sodium pumps, disrupt cellular function and lead to electrolyte imbalances and decreased cardiac output despite advancements in burn care, challenges [96], such as skin damage, pain management, infection control, slow wound healing, fluid loss and selecting appropriate wound dressings persist [58].

CHs emerge as a promising material for achieving these dressing requirements. For example, a hydrogel with superior cooling capacity has been developed through a 3D heat transfer network that retains wound moisture, absorbs exudate, promotes angiogenesis and maintains electrical gradients through ES to promote wound healing [40]. Its efficacy was further validated in a rat dorsal scald model, demonstrating reduced thermal injury, mitigated inflammatory responses and accelerated wound healing. On the other hand, frostbite often occurs due to prolonged exposure to low temperatures, triggering vasoconstriction. Prolonged exposure or extremely low temperatures can form ice crystals in intracellular and extracellular fluids. Thawing can further exacerbate damage, manifesting as vasodilation, congestion, exudation and thrombosis. Ice crystals can cause cell dehydration, protein denaturation, decreased enzyme activity and tissue necrosis. Notably, combined a PPHM hydrogel epidermal sensor with a miniature analog-to-digital converter chip, resulting in a novel wound dressing (Figure 7C) [90]. This dressing accelerated wound healing by absorbing excess exudate and exhibited high sensitivity for monitoring physiological signals. Experimental results confirmed the hydrogel's efficacy in promoting wound healing and its real-time monitoring capabilities in low-temperature environments, providing a comprehensive overview of the healing process.

### Intelligent CHs for athletic joint wounds with movement monitoring capabilities

Upon joint injury, the healing process of wounds tends to be sluggish. This sluggishness is primarily attributed to the unavoidable skin stretching, frequent joint movements and bending associated with locomotion and exercise [97]. These activities and pressure at the joint site continuously perturb the healing process, exerting additional stress on the wounded tissue. Consequently, this may result in the stretching and displacement of wound margins, thereby impeding the healing progression. Additionally, wounds on mobile regions are predisposed to secondary tears and dressing dislodgment. The proximity of bones and soft tissues further exacerbates the risk of bacterial infestation. External mechanical stresses can also contribute to the wound dressings' deformation, wear or damage. In this context, self-healing hydrogels exhibit remarkable potential. These hydrogels can self-repair upon damage, thereby prolonging the lifespan of the dressing, affording more robust wound protection and facilitating healing. Furthermore, hydrogel dressings endowed with strain-sensing abilities can monitor the dressing's status and mitigate complications such as excessive absorption, swelling, ruptures and severe deformations that may arise during the healing of wounds on mobile regions. The CHs exhibited commendable flexibility, self-healing properties, antibacterial activity and tissue adherence (Figure 7D) [31]. When applied to wounds on moving joints, such as fingers, elbows or wrists, the hydrogel's resistance varied sensitively and consistently with joint movement. Upon full joint extension, the resistance returned to its baseline value. As a result, these CHs served as adept sensors for human body movements, facilitating sensitive and stable monitoring of diverse activities with swift responsiveness. This innovative approach significantly reduced the likelihood of complications, including excessive absorption, swelling, ruptures and severe deformations, during the healing process of wounds on mobile joints.

## Multifunctional intelligent CHs and their diverse applications

In personalized strategies for chronic wound management, traditional treatments for diabetic ulcers, infected wounds, extreme temperature injuries and athletic joint wounds have shown gradual limitations in addressing the special needs of these wounds. To promote the healing of these complex wounds more effectively, researchers are constantly exploring innovative techniques. In recent years, the utilization of multifunctional CHs is progressively evolved, converging with advancements in deep learning and flexible electronics. This convergence enables their extended application in diverse contexts, such as self-powered systems, electrically-triggered drug delivery, comprehensive diagnostic and therapeutic approaches, and scar-free healing. In contrast to the cumbersome and inflexible nature of traditional electronics, wearable devices exhibit streamlined operation, versatility, affordability and lightness, thereby improving patient adherence [72]. These gadgets facilitate focused and controlled interventions without disrupting the patient's routine activities. By amalgamating leading-edge technologies, such as machine learning and advanced sensor systems, the prospects of intelligent wound dressings in wound management are continually broadening [89].

### Intelligent wound dressing with self-powered dressings

Notably, the self-powered portable CHs wound dressing emerged as a groundbreaking variety, merging self-powered technology with CHs [98]. This dressing employs specific methods, including thermoelectricity generation, piezoelectricity and triboelectric nanogenerators, to transform energy derived from bodily movements or temperature fluctuations into electrical energy. This electricity powers the CHs, eliminating the need for an external power source and ensuring uninterrupted wound treatment and care.

Consequently, it expedites wound healing and is well-suited for direct application on the human body. Integrated CHs with a triboelectric nanogenerator operating in contact-separation mode, efficiently harnessing the body's biomechanical energy [99]. The electrostatic effect triggered by movement-induced gaps stimulates ion movement within the hydrogel, balancing the electrostatic charge. This polarization established an electric layer at the hydrogel interface, generating an equal distribution of negative and positive charges within the metal wire. As the separation distance increased, the potential difference between the interfaces widened, prompting electron flow from the hydrogel to the body. An EF between the triboelectric patch and human tissue ultimately accelerated the healing process. As materials science continues to evolve, researchers will likely develop CHs materials with superior conductivity, biocompatibility and adaptability, as well as cutting-edge energy conversion technologies. These innovations are expected to support the implementation of wireless, low-power energy harvesting and storage systems within hydrogel-based intelligent bandages. As a result, this will increase the efficacy and safety of wound dressings, enable more durable power delivery and reduce dependence on external power sources. Ultimately, these advances will meet the demands for intelligence, integration and portability in wound management.

### Intelligent wound dressing with electrically-triggered drug delivery systems

CHs can be equipped with the ability to facilitate the controlled delivery of antibiotics, antimicrobial agents, cells, cytokines and other substances, thereby enhancing real-time monitoring and control of the wound environment. These hydrogels, designed for electrically stimulated drug release, can adjust adaptively to the specific conditions of the wound, conforming precisely to its contours and ensuring targeted drug delivery to the affected region. Furthermore, CHs-based intelligent wound dressings are capable of adjusting the release of encapsulated therapeutic agents or bioactive compounds in response to changes detected by ES. This optimization boosts drug permeability and bioavailability, subsequently expediting the wound-healing process. Wan and colleagues [89] innovated an advanced heterogeneous interface intelligent CHs sensor formulated with PPY@PDA/PANI/PVDF. This sensor possessed the capability to learn the ammonia response unique to individual wounds adaptively and regulated the drug release rate of the PPY@PDA/PANI hydrogel under electrical cues. This technology encompassed monitoring, self-diagnosis and adaptive adjustment functionalities, fully realizing intelligent drug delivery and tailored treatment approaches for complex wound management. Further, these CHs utilized for electrically stimulated drug release hold potential for integration with bioelectronic devices, enabling remote surveillance and meticulous manipulation, ultimately elevating treatment convenience, effectiveness and patient comfort.

### Intelligent wound dressing with comprehensive diagnostic and therapeutic approaches

Due to their superior elasticity and stability, CHs prevent damage or discomfort upon contact with host organisms. This unique characteristic enables extended and in-depth observation of intricate physiological parameters, along with real-time monitoring. These features are especially advantageous for patients requiring continuous monitoring, thus greatly aiding in the diagnosis, treatment and comprehensive management of various diseases [100]. The CHs dressing for comprehensive diagnostic and therapeutic approaches seamlessly blend diagnostic and therapeutic technologies with AI. This dressing leverages the exceptional properties of CHs and aligns with comprehensive diagnostic and therapeutic approaches, striving for intelligent and tailored wound management. By incorporating sensors and a drug delivery system, this dressing can continuously track wound physiology, including temperature, pH and humidity, relaying this critical information to medical professionals or patients. Physicians can utilize this data to tailor treatment strategies, such as adjusting drug release rates or types, to meet individual patient needs. This dressing dynamically adjusts ES based on the wound's healing progress to optimize healing outcomes. Point-of-care testing (POCT) also plays an important role in hydrogel synthesis [101]. For POCT considerations, a hydrogel capable of rapid synthesis in 10 s was prepared [102]. This hydrogel's strain-sensing capability paved the way for innovative point-of-care diagnostics, ushering in a new era of swift and accurate medical response.

Despite advances, intelligent wound dressings still face challenges in data accuracy, reliability and sustained drug release and ES therapy. Advances in the Internet of Things (IoT), big data and AI will enhance these dressings with more sensors and intelligent algorithms, enabling seamless real-time wound monitoring, cloud data synchronization and precise individualized treatments, thus integrating diagnosis and therapy.

### Intelligent wound dressing with scar-free healing

Scarring, which results from excessive collagen deposition and myofibroblast growth, is a prevalent outcome of skin injury and is collectively termed scarring [103]. This scarring can impose substantial physiological and psychological burdens on adult patients. Factors contributing to excessive scarring encompass genetic predisposition, hypertension, endocrine disorders, autoimmune diseases and hormonal shifts [104]. The intricate wound healing and scar formation process involves numerous molecular, biological and mechanical components. While current clinical approaches, including surgical resection, thermal laser therapy, gene therapy and drug administration, have yielded some success in scar treatment, they often fall short of eliminating scars.

Interestingly, wound dressings with additional conductive properties can effectively stimulate the migration of healthy cells to the wound site, thereby accelerating blood flow, inhibiting bacterial growth and enhancing tissue oxygenation. Simultaneously, the rapid proliferation of healthy cells restricts the formation of fibrous tissue and scars, ultimately achieving scar-free healing. Liu and colleagues [105] recently developed a hydrogel characterized by its high strength, toughness, anti-swelling, antibacterial and antioxidant properties. Utilizing an infection model, they implemented targeted interventions based on the various stages of wound healing. The findings revealed that controlling bacterial infection was more effective in anti-scarring efforts than reducing oxidative stress. Currently, most studies focus on minimizing scar formation by expediting wound closure. However, the efficacy of a standalone hydrogel in scar prevention is limited, suggesting that incorporating diverse components could further enhance its scar-free healing potential. Nonetheless, research in this domain still needs to be advanced. Furthermore, the physiological mechanisms and practical applications of scar inhibition through ES require deeper exploration, and the impact of various ES modes on fibroblasts remains to be further investigated.

## Challenges and outlook

With rapid advances in materials science and tissue engineering, coupled with a growing interest in cutaneous wound management and overall health management, CHs have emerged as a key research area for the development of intelligent wound dressings. Their unique properties in terms of wound healing and monitoring capabilities have attracted considerable attention from researchers. However, they face challenges such as material stability, conductivity and biocompatibility balance. Currently, CHs must cope with complex wound environments and maintain their structural and functional stability, while ensuring uniform current distribution to promote cell proliferation and migration. In addition, the material design must balance conductivity and biosafety to avoid triggering an immune response or interfering with the wound-healing process.

To overcome these difficulties, future research should focus on innovative material design, such as the development of adaptive CHs that dynamically respond to changes in the wound and optimize the current conduction path. At the same time, intelligent regulation mechanisms should be introduced to automatically adjust the ES parameters according to the wound-healing stage to achieve personalized treatment. In addition, interdisciplinary cooperation combining biomedical engineering, materials science and AI should be strengthened to promote the in-depth application of CHs in the field of wound repair.

In addition, CHs may be integrated with AI in the future, providing innovative solutions for precise sensing and comprehensive health monitoring. These CHs can mimic the human senses, enabling tactile [106], olfactory [107], auditory [108], gustatory [109], visual [110] and physiological sensing [111] with high precision. As stress-strain sensors, CHs can measure mechanical stress and strain, yielding valuable data for applications ranging from prosthetics [112] to human–machine interfaces [32]. The next-generation AI-powered CHs for data collection and analysis, and the upcoming intelligent wound dressings will seamlessly integrate diagnosis, treatment, monitoring and inspection, enabling personalized health management tailored to chronic wound patients [113, 114]. This innovation will significantly improve the medical experience and satisfaction of patients while alleviating the workload of clinicians.

However, several major bottlenecks remain, including high-quality collection of wound intelligence information, the establishment of personal medical data platforms, secure transmission of disease and medical data and generalized applications.

The acquisition of high-fidelity wound data represents a significant challenge for intelligent wound dressings that leverage CHs, as this is an essential component of their sensing-based intelligence. The dynamic nature of wound care data, which is voluminous and subject to rapid changes, can impede the training of AI models. To ensure effective monitoring, these dressings must collect real-time, precise wound data, with AI algorithms providing immediate labeling and analysis. Furthermore, maintaining stable contact with the skin to prevent signal loss during movement is of paramount importance [115].

The generation of complex data sets by intelligent wound dressings based on CHs necessitates the implementation of sophisticated data handling and integration strategies. To address this issue, it is essential to implement a cloud-connected diagnostic platform for real-time data acquisition and analysis, which should be complemented by multimodal sensing to improve accuracy. The processing of this data requires the use of high-performance computing and storage solutions. In light of the constraints of existing power solutions, future research should prioritize the development of flexible, low-power energy systems for CH-based dressings, to ensure patient comfort and practicality [116].

The pervasiveness of AI is overshadowed by persistent concerns surrounding data access, ownership and security. The absence of a unified regulatory framework to address these issues represents a significant challenge [117]. The extent to which users are willing to share sensitive medical data to train AI models is uncertain, particularly in light of the privacy protocols that govern access to medical data. As AI in medicine progresses, it is essential to prioritize data protection and transparency to maintain public trust and ensure beneficial outcomes [118].

The use of CHs as intelligent wound dressings represents a new advancement in AI technology, flexible electronic materials and healthcare. In conclusion, AI-powered CHs represent a transformative approach to life health, enabling precise sensing, comprehensive data integration and advanced health monitoring solutions. The ongoing research and development in this field promise significant advancements in medical technology and patient care.
